# Development of standard operating protocol for measurement of cassava root mealiness

**DOI:** 10.1038/s41598-024-68441-4

**Published:** 2024-07-27

**Authors:** O. A. Osunbade, E. O. Alamu, W. Awoyale, M. Adesokan, B. A. Akinwande, J. A. Adejuyitan, B. Maziya-Dixon

**Affiliations:** 1 International Institute of Tropical Agriculture (IITA), Oyo Road, PMB 5320, Ibadan, Oyo State Nigeria; 2 Department of Food Science, Ladoke Akintola University of Technology, PMB 4000, Ogbomoso, Nigeria; 3 International Institute of Tropical Agriculture, Southern Africa, Research and Administration Hub (SARAH) Campus, P.O. Box 310142, Chelstone, Lusaka 10101 Zambia; 4 Department of Food Science and Technology, Kwara State University, P. M. B 1530, Malete, Kwara State Nigeria

**Keywords:** Softness, Chewiness, Extrusion, Hardness, Cooking time, Biological techniques, Plant sciences

## Abstract

One of the major attributes of boiled cassava roots is its ability to soften within a short period, otherwise known as mealiness. This study aimed to establish and validate standard operating procedures for assessing the mealiness of boiled cassava roots. Twenty cassava genotypes, including landrace and improved varieties, were selected for the protocol development, with an additional ten genotypes used for validation. Following cooking, the cassava roots were evaluated for hardness and work done in extrusion using a texturometer equipped with a five-blade Ottawa cell probe. The same samples were assessed for sensory texture analysis using trained panelists for parameters such as softness and chewiness. Pearson's correlation analysis revealed significant positive correlations (p < 0.01) between sensory softness and instrumental texture measurements, as well as between softness and cooking time (p < 0.01, r = 0.94), and between chewiness and cooking time (p < 0.05, r = 0.81). Validation results confirmed significant correlations (p < 0.01) between cooking time, sensory softness, and chewiness. These findings suggest that cooking time can serve as a reliable indicator, closely associated with sensory attributes, in determining the mealiness of boiled cassava roots. This approach offers a practical, mid-throughput method for assessing cassava root mealiness, with implications for breeding improved varieties, farmers adoption, and consumer acceptance.

## Introduction

Cassava (*Manihot esculenta* Crantz) is a significant stable food in tropical regions, including developing countries in Africa. It accounts for more food calories per unit weight when compared to yam. Due to its effective energy production, year-round availability, resistance to extreme climatic conditions and compatibility with Africa’s current farming and food systems, cassava contributes to resolving the current continent’s food challenges^1^. About 278 million metric tons of cassava are produced globally, with Nigeria producing over 63 million metric tons^2^. Nigeria was followed in production by Thailand (31 million metric tons), Brazil (24 million metric tons), Indonesia (21 million metric tons), Ghana (18 million metric tons), and other countries with similar numbers. Cassava is grown mainly in the states of Imo, Anambra, Benue, Kogi, Enugu, Ogun, Ondo, Taraba, Delta, and Osun, while it is also grown in smaller quantities in other states of Nigeria^3^. New cassava varieties must have end-user culinary qualities, as farmers attach equal value to agronomic performances and end-user culinary quality traits^4^.

In Nigeria, breeding efforts focus on improving cassava root cooking and eating qualities, predominantly texture such as mealiness, to address the increasing demand for varieties suitable for the fresh consumption market segment. In Nigeria, cassava roots have been processed into many fermented and unfermented products, and some fermented products include *lafun*, *gari*, fufu and *pupuru*^5^. High-quality cassava flour (HQCF) is a typical type of unfermented cassava product^5^. Other unfermented products include pasta, Abacha, snacks and cassava chips, amongst others^6^. Consumers’ acceptance of cassava genotypes largely relies on their agronomic performance and end-user culinary qualities such as root mealiness. Mealiness is a term used to describe the boiled cassava root that dissolves easily in the mouth upon biting. It also describes cassava roots’ softness and chewability after being boiled^7^. Consumers have considered it the most essential attribute of boiled cassava roots. It is important to elicit the descriptors of mealiness from the consumer’s perspective, and consumer preference is often determined using the degree of liking and measured using hedonic scales. Although panelists can be asked to indicate their degree of liking, preference, or acceptance of a product directly, hedonic tests are often used to measure preference or acceptance indirectly. Category scales, ranking tests and the paired-comparison test can all be used to assess product acceptance.

Recently, the Just-About-Right (JAR) test and Check-All-That-Apply (CATA) tests were developed to complement the results of the hedonic test^8^. The JAR question format seeks to determine the optimum intensity of a sensory attribute by asking consumers if they consider it too strong, too weak or just-about-right (JAR)^9^. The CATA test describes the products with all the descriptors that best describe the products^10^. The CATA, also known as “choose-all-that-apply”, is a question format used in recent years to obtain rapid consumer product profiles. Consumers are presented with attributes and asked to indicate which words or phrases appropriately describe their experience with the evaluated sample^11,12^.

Some cassava varieties can be boiled and consumed directly alone or with palm oil, and vegetable oil because they are mealy. Mealy roots cook faster and are soft and easy to chew. These parameters can be measured by textural instrumental techniques and sensory texture analysis to establish a protocol for the mealiness of cassava. However, previous studies have provided information on the texture of boiled cassava roots and their mealiness using penetration tests^13,14^, but there were no details on the descriptors of mealiness, especially by the consumers. This research intends to develop and validate a standard operating procedure (SOP) for measuring boiled cassava root’s mealiness using correlation coefficients between cooking time and the sensory and instrumental textural attributes.

## Materials and methods

### Materials

Twenty (20) cassava genotypes (landraces and improved) between the age of 10–12 months were freshly harvested from the International Institute of Tropical Agriculture (IITA) research farm. These include: TMS16F2021P0044, TMS16F2022P0057, *ege funfun*, *ege dudu*, IBA961632, TMEB711, IBA011797, TMEB419, TMS16F2021P0011, IBA184416, IBA184433, IBA184427, IBA184429, IBA184445, IBA30572, IBA184422, IBA184449, IBA184439, IBA070593 while TMEB693 being the last used as check. Other materials and tools such as an aluminum steamer, texture analyzer, five-blade Ottawa grid cell, infrared thermometer, weighing scale, plastic bowl, aluminium foil and stopwatch were sourced from Food and Nutrition Sciences Laboratory (FNSL), IITA, Ibadan, Nigeria.

### Methods

#### Product preparation for analysis

**Figure 1:**
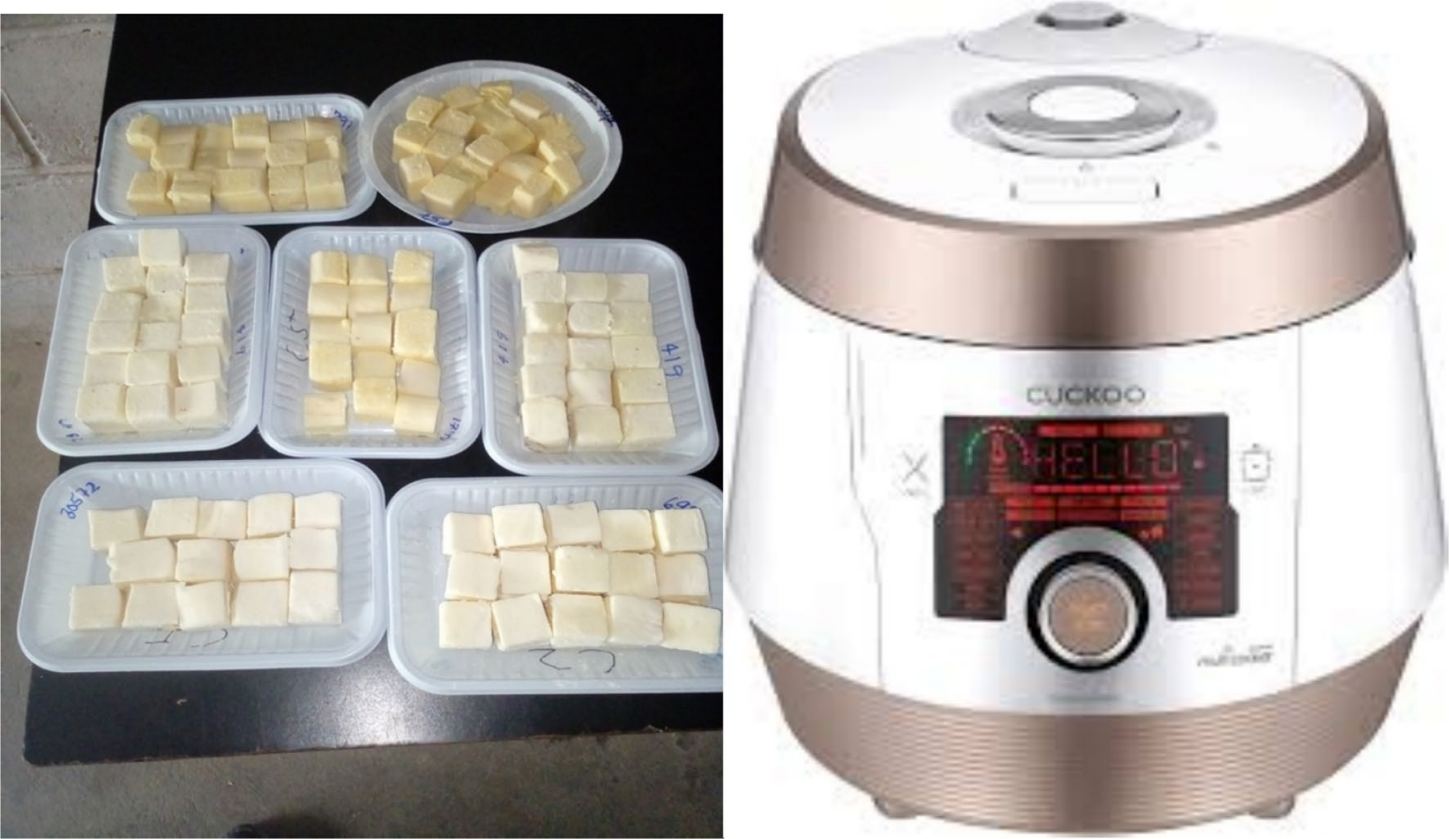
A picture showing cassava root cubes and a Steam cooker. Source: Maziya-Dixon et al. (2023) (Next Gen report).

Freshly harvested cassava roots were washed, peeled, and diced using a 2.5 × 2.5 cm stainless steel punch to produce cubic-shaped pieces of similar dimensions from central parts of the root (Fig. 1). The samples were steam-cooked using a steamer (Fig. 1) until a fork penetrated softly. Six (6) measurements were taken for each sample and cooked cassava cubes were wrapped in aluminium foil and transferred into a food warmer to minimize physical changes due to cooling. The sample temperature was monitored using an infrared thermometer. Similar sample preparation procedures and a temperature of 45 °C were used for instrumental and sensory texture analysis.

#### Determination of cassava roots’ mealiness

**Figure 2:**
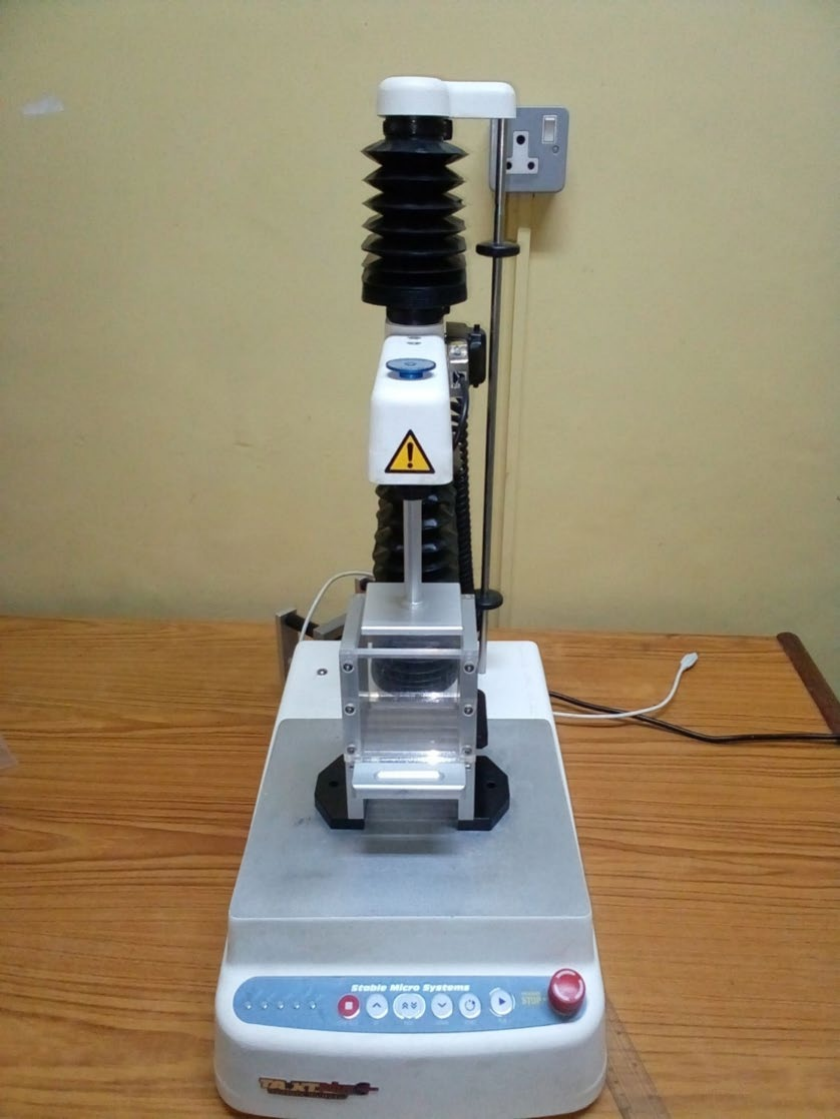
A picture of the texture analyzer. Source: Maziya-Dixon et al. (2023) (Next Gen report).

The methods to measure cassava root mealiness can be divided into sensory texture analysis (subjective) and instrumental texture analysis (objective). Fifteen trained panellists were used for the sensory texture analysis of the cooked cassava roots, and the performances of each panellist were tested for repeatability. The same batch of steam-cooked cassava root was subjected to instrumental measurement using a texturometer (Fig. 2) to determine the mechanical force required and the work done during extrusion^15^. The panellists were trained on two primary sensory descriptors for the mealiness of boiled cassava as determined from consumers’ acceptability study (i.e., softness and chewiness). Staffs and students that are familiar with boiled cassava roots were selected at IITA, trained on the descriptors of boiled cassava roots, validated and screened based on their performance and ability to generate repeatable results.

#### Sensory texture analysis (subjective)

Among the simplest subjective methods used to evaluate the degree of softening are pressing between fingers, biting in the mouth, and checking the easiness of chewing. Cassava roots were cleaned, peeled and manually punched into about 2.5 × 2.5 cm long cubes and subjected to steam-cooking until the fork penetrated easily to measure cooking time (CT). The panellists evaluated the samples on the scale of 1–3 to rate the softness (1 = soft; 2 = moderately soft; 3 = hard) and chewiness (1 = chew easily; 2 = chew moderately; 3 = hard to chew).

#### Instrumental texture analysis (hardness and workdone)

The instrumental texture measurements of the boiled cassava roots were carried out using a Texture Analyser (Model TA-XTplus, Stable Micro System, Haslemere, U.K) coupled with an extrusion probe (Ottawa five-blade grid) (Fig. 2). The texture parameters, such as hardness and work during extrusion, were measured in six replications^15^.

### Statistical analysis

The data obtained from cooking time, chewiness, softness, hardness and workdone were analyzed for correlation coefficients using Statistical Package for Social Sciences (SPSS version 16.0) and R-language software.

### Declaration on research involving plants

This is to declare that all the materials and methods used in this research are in accordance to institutional standards.

### IUCN policy statement on research

Collection of plant materials for experimental research were in-line with institutional, national, and international guidelines and legislation.

## Results and discussion

### Cooking time, sensory and instrumental texture analysis

The steaming method was used for cooking time evaluation, and results showed that some varieties had shorter cooking times between 10 and 15 min (TMEB 693 and TMEB 419). Some cassava varieties also had medium cooking time between 17 and 20 min (IBA011797, IBA30572 and TMS16F2022P0057), and TMS16F2021P0044 had cooking time above 20 min. The sensory panellist’s scores for the cassava varieties in softness ranged from moderately soft to hard and chewiness ranged from “easy to chew” to “hard to chew”. Instrumental hardness ranged from 1367.0 to 2946.7 g, while instrumental work during extrusion ranged from 1935.8 to 7900.6 g/s (Table 1).

**Table 1:** Sensory and instrumental texture attributes, and cooking time of boiled cassava roots.

Cassava roots	Sensory texture attributes		Instrumental texture attributes*		Cooking time (min)
	Chewiness	Softness	Hardness (g)	Work done (g/s)	
TMEB 419	1.40^f^	1.90^e^	2946.70^a^	2250.88^e^	15^e^
TMEB 693	1.50^e^	1.50^f^	1367.00^f^	1935.88^f^	10^f^
TMS16F2021P0044	2.30^b^	3.50^a^	2682.60^c^	7900.64^a^	21^a^
IBA 30572	2.10^c^	2.80^c^	2164.00^d^	4870.31^b^	20^b^
TMS16F2022P0057	2.50^a^	3.00^b^	2767.40^b^	4114.63^c^	19^c^
IBA 011797	2.00^d^	2.30^d^	2029.00^e^	2821.65^d^	17^d^

*Texture results are an average of six measurements.

Mean values with the same super script along the column are not significantly different.

Key: chewiness: 1 = easy to chew, 2 = moderately chew, 3 = hard to chew; Softness: 1 = soft, 2 = moderately soft, 3 = hard.

### Pearson’s correlation between instrumental texture profiling and sensory analysis of boiled cassava

Results of Pearson’s correlation showed that a significant (p < 0.01) and positive correlation (r = 0.92) exists between the sensory softness and the instrumental work done by extrusion (Table 2). There was no significant correlation between instrumental hardness and sensory texture attributes. Also, there was a significant positive correlation between sensory softness and cooking time (p < 0.01, r = 0.94) and between sensory chewiness and cooking time (p < 0.05, r = 0.81).

**Table 2:** Pearson’s correlation coefficient for experimental data.

	Work done (g/s)	Cooking time (min)	Chewiness	Softness
Hardness	0.39 ns	0.61 ns	0.32 ns	0.54 ns
Work done (g/s)		0.81 ns	0.70 ns	0.92**
Cooking time (min)			0.81*	0.94**
Chewiness				0.89*

*Correlation is significant at the 0.05 level.

**Correlation is significant at the 0.01 level.

### Validation of the developed standard operating procedure (SOP)

Ten cassava varieties were included to validate the protocols, and new measurements were conducted using the same protocols. The roots were washed, peeled and diced into cubes using a punch of 2.5 × 2.5 cm long, washed, and steam-cooked until the fork penetrated the boiled cassava easily according to the developed step-by-step procedure to measure mealiness. The sensory evaluation by 15 trained panellists was conducted concurrently with instrumental texture profiling analysis. Statistical analysis was conducted using SPSS and XLSTAT. The correlation coefficients were significant at 0.01 between cooking time, sensory softness, and chewiness (Table 3).

**Table 3:** Pearson’s correlation coefficient from validation data.

	Work done (g/s)	Cooking time (min)	Chewiness	Softness
Hardness	0.49 ns	0.21 ns	0.22 ns	0.16 ns
Work done (g/s)		0.30 ns	0.26 ns	0.28 ns
Cooking time (min)			0.98**	0.85**
Chewiness				0.89**

*ns* not significant difference.

**Correlation is significant at the 0.01 level.

### Critical points and notes on the procedure

(i) Analysis of the sample needs to be done very quickly, between 10 and 20 min, as the texture of boiled cassava roots changes significantly with time when cooling.
(ii) The same serving temperature (40–45 °C) must be maintained for instrumental and sensory analysis.
(iii) The trigger force of the compression probe must be carefully adjusted to forestall the total collapse of the sample at the first compression cycle.
(iv) The sensory and instrumental texture analysis must be conducted at the same time with the same serving temperature to correlate sensorial and instrumental texture results.
(v) Each sample must be replicated in a minimum of six times to obtain an actual representative.

## Conclusion

This study has attempted to develop a SOP to measure the mealiness of boiled cassava roots. Sensory and instrumental texture measurements were conducted concurrently on selected cassava genotypes. Trained panellists were used for the sensory texture analysis, and the descriptors for mealiness were softness and chewiness; these were obtained from a previous study (unpublished), which involved about 120 respondents in a community where boiled cassava is widely consumed. Findings showed that no instrumental parameters directly measured mealiness, as no significant correlations were established between instrumental hardness and softness or chewiness of boiled cassava roots. Work done during extrusion significantly correlates with softness during the experiment’s first phase, but no significant difference was observed during the validation stage. However, cooking time, considered a mid-throughput method, significantly correlates with both softness and chewiness and a similar trend was observed after validation. Therefore, cooking time could be considered a mid-throughput method for determining the mealiness of boiled cassava. It is recommended that more diverse cassava populations be used to repeat this experiment to establish a correlation between the instrumental measurement and the mealiness of boiled cassava.

## Data Availability

Data is available on request. If any one want to request of data on this work, he or she should contact the lead author or the corresponding author.
